# Littermate-Controlled Experiments Reveal Eosinophils Are Not Essential for Maintaining Steady-State IgA and Demonstrate the Influence of Rearing Conditions on Antibody Phenotypes in Eosinophil-Deficient Mice

**DOI:** 10.3389/fimmu.2020.557960

**Published:** 2020-10-15

**Authors:** Rachael D. FitzPatrick, Mia H. E. Kennedy, Katherine M. Lawrence, Courtney M. Gauthier, Brandon E. Moeller, Andrew N. Robinson, Lisa A. Reynolds

**Affiliations:** Reynolds Laboratory, Department of Biochemistry and Microbiology, University of Victoria, Victoria, BC, Canada

**Keywords:** eosinophils, secretory IgA, circulating IgA, antibodies, co-rearing, co-housing, littermate controls

## Abstract

Conflicting data has emerged regarding a role for eosinophils in IgA production, with some reports that eosinophils support both secretory and circulating IgA levels during homeostasis. Previous studies have compared antibody levels between wildtype and eosinophil-deficient mice, but these mice were obtained from different commercial vendors and/or were not littermates. Thus, the possibility remains that extrinsic environmental factors, rather than an intrinsic lack of eosinophils, are responsible for the reports of reduced IgA in eosinophil-deficient mice. Here we used wild-type and eosinophil-deficient (ΔdblGATA) mice that were purchased from a single vendor, subsequently bred in-house and either co-housed as adults, co-reared from birth or raised as littermates. We found no differences in the levels of secretory IgA or in the numbers of small intestinal IgA-producing plasma cells between wild-type and ΔdblGATA mice, demonstrating that under controlled steady-state conditions eosinophils are not essential for the maintenance of secretory IgA in the intestinal tract. While we found that levels of IgM and IgE were significantly elevated in the serum of ΔdblGATA mice compared to co-reared or co-housed wild-type mice, no significant differences in these or other circulating antibody isotypes were identified between genotypes in littermate-controlled experiments. Our results demonstrate that eosinophils are not required to maintain secretory or circulating IgA production and the absence of eosinophils does not impact circulating IgG1, IgG2b, IgM, or IgE levels during homeostasis. These findings emphasize the importance of optimally controlling rearing and housing conditions throughout life between mice of different genotypes.

## Introduction

Historically, eosinophils have been regarded as type 2 effector cells during both helminth infection and allergic inflammation (1–5). During homeostasis, eosinophils reside in the thymus, uterus, bone marrow and the gastrointestinal tract and in contrast to in type 2 immune settings, their steady-state function within these sites is not well understood (6). Notably, under steady-state conditions eosinophils comprise up to 25% of all leukocytes in the small intestinal lamina propria (LP) (7) and recent data suggests that eosinophils play a key role in several homeostatic processes within the intestinal tract (8). For example, eosinophil-deficient mice have been reported to have impaired mucus production (9), stunted development of Peyer's patches (9) and a unique bacterial microbiota composition (9–11). In addition, two independent studies have reported a strict requirement for eosinophils in the maintenance of IgA-producing plasma cells in the small intestine with reduced secretory and serum IgA levels found in eosinophil-deficient mice (9, 10). Despite these findings, other studies have reported no difference in secretory or serum IgA levels between wild-type and eosinophil-deficient mice (7, 11) and recently, reported differences in IgA levels between wild-type and eosinophil-deficient mice have been attributed to bacterial microbiota compositional differences rather than due to an intrinsic absence of eosinophils (12). Similar contradictions in published literature exist regarding the function of homeostatic eosinophils in the bone marrow: an initial report found that eosinophils are required for bone marrow-resident plasma cell survival (13), while later studies have demonstrated that eosinophils are not required to carry out this function (14, 15).

Previous studies that have compared IgA levels between wild-type and eosinophil-deficient mice have used a variety of housing and breeding schemes, varying from no co-housing and genotypes sourced from different vendors to different genotypes raised as littermates. We postulated that the conflicting reports in the contribution of eosinophils to the maintenance of IgA production could be explained by variations in the intestinal microbiota driven by differences in environmental housing conditions of genetically-modified vs. wild-type mice (extrinsic effects), rather than genotype-driven changes in immune functions and microbiota composition (intrinsic effects). Indeed, constituents of the bacterial microbiota do have the capacity to alter both secretory IgA (sIgA) and systemic antibody levels (12, 16–20).

In the present study, we aim to address this issue using BALBc/J wild-type and eosinophil-deficient (ΔdblGATA) mice (21) that were purchased from a single vendor and subsequently bred in-house. We either co-housed wild-type and eosinophil-deficient mice as adults, co-reared them from birth or generated wild-type and ΔdblGATA littermate controls. Under these conditions we found no difference in the levels of intestinal sIgA or numbers of small intestinal IgA-producing plasma cells between wild-type and ΔdblGATA mice. We also found no deficiency in circulating IgA in ΔdblGATA mice. While levels of circulating IgA, IgM, and IgE were in fact elevated in the serum of ΔdblGATA mice that were co-reared or co-housed with wild-type mice, no differences in the levels of these antibody isotypes were found between littermate wild-type and ΔdblGATA mice. Our results demonstrate that eosinophils are not essential for the maintenance of serum or secretory antibody levels during homeostatic conditions. These findings emphasize the importance of carefully controlling for extrinsic effects during development when comparing antibody levels between mice of different genotypes.

## Materials and Methods

### Mice

All mouse experiments were performed at the University of Victoria (UVic), approved by the UVic Animal Care Committee and followed all guidelines set by the Canadian Council on Animal Care. Wild-type BALB/cJ and ΔdblGATA BALB/cJ mice were purchased from The Jackson Laboratory and subsequently bred in-house prior to use in experiments. Mice had access to food and water *ad libitum* and were housed in individually ventilated cages under specific pathogen-free conditions. Female wild-type and ΔdblGATA mice born in separate cages to wild-type and ΔdblGATA dams respectively were co-housed when they were 6–11 weeks old and were euthanized 1 week later for sample collection (Supplementary Figure 1A). “Co-reared” or “littermate” wild-type and ΔdblGATA male mice were also generated. “Co-reared” mice were generated by setting up breeding trios consisting of one ΔdblGATA male with one ΔdblGATA female and one wild-type female in a single cage. Male mice born to the ΔdblGATA female in this cage were all of the ΔdblGATA genotype, and male mice born to the wild-type female in this cage were all of the wild-type genotype. Co-reared male mice continued to be housed together after weaning, and were euthanized for sample collection when they reached 6–12 weeks old (Supplementary Figure 1B). “Littermate” male mice were generated by setting up breeding trios consisting of one ΔdblGATA male with two females heterozygous for the ΔdblGATA mutation. Male mice born to these heterozygous females were either of the wild-type or ΔdblGATA genotype. Littermate male mice continued to be housed together after weaning, and were euthanized for sample collection when they reached 6–12 weeks old (Supplementary Figure 1C). The dblGATA mutation is X-linked (21) so it is not possible to generate wild-type and homozygous ΔdblGATA female offspring in the same cage from either the co-rearing or littermate control breeding scemes, therefore female mice were not used in these experiments.

### Genotyping PCR

DNA was extracted from ear clips digested with 25 mM NaOH/0.2 mM EDTA with heating at 98°C for 1 h. Samples were neutralized with 40 mM Tris HCl and centrifuged at 4,000 rpm for 3 min. Supernatants containing extracted DNA were used for PCR using Taq DNA Polymerase (New England Biolabs Inc.). To distinguish between wild-type and ΔdblGATA alleles the primers G1mutF1: 5′-CCCAATCCTCTG-GACTCCCA-3′, and G1mutR: 5′-CCTACTGTGTACCAG-GCTAT-3′ (21) were used which amplify a 509-bp region from ΔdblGATA mice and a 459-bp region from wild-type mice (Supplementary Figure 2). Samples were run on a PxE 0.2 Thermal Cycler (Thermo Electron Corporation), under the following cycling conditions: 1 cycle at 94°C for 2 min; 35 cycles of 94°C for 30 s, 57.5°C for 30 s, 72°C for 30 s; 1 cycle at 72°C for 5 min. A 1.5% agarose gel with SYBR safe DNA gel stain (Invitrogen) was used to visualize the PCR products.

### Quantification of Antibody Levels by ELISA

An Enzyme-Linked Immunosorbent Assay (ELISA) was used to quantify antibody levels in either sera, small intestinal lavages or feces. To collect small intestinal content the entire small intestine was dissected and then lavaged with 1 mL PBS. Blocking solution (PBS + 2% BSA) was added to fecal pellets at a concentration of 100 mg feces/mL. Both feces and small intestinal lavages were homogenized using a Bead Mill 24 (Fisher Scientific) and centrifuged at 10,000 rpm for 10 min to collect the supernatant. Intestinal content and fecal supernatants were stored at −20°C prior to analysis. Blood samples were left to clot for 3–6 h at 4°C prior to centrifugation to isolate the serum. Serum samples were stored at −80°C prior to analysis. NUNC maxisorb flat-bottom 96-well plates (ThermoFisher) were coated with the following antibodies (all from BD Biosciences) diluted to 1:250 in 0.1 M sodium carbonate (pH 9.5): rat anti-mouse IgA (Clone C10-3), rat anti-mouse IgG1 (Clone A85-3), rat anti-mouse IgG2b (Clone R9-91), rat anti-mouse IgM (Clone II/41) or rat anti-mouse IgE (Clone R35-72) and left at 4°C overnight. Plates were blocked for 2 h at 37°C with blocking solution. A dilution series of each sample was incubated on plates overnight at 4°C alongside a standard curve of purified recombinant mouse IgA, IgG1, IgG2b, IgM or IgE (BD Biosciences). The following primary antibodies (all from BD Biosciences) were diluted to 1:1,000 in blocking solution: biotin rat anti-mouse IgA (Clone C10-1), biotin rat anti-mouse IgG1 (Clone A85-1), biotin rat anti-mouse IgG2b (Clone R12-3), biotin rat anti-mouse IgM (Clone CR6-60.2) or biotin rat anti-mouse IgE (Clone R35-118) and incubated on plates for 1 h at room temperature. Plates were then incubated with streptavidin-HRP (BD Biosciences) diluted to 1:1,000 in blocking solution for 1 h in the dark at room temperature prior to developing the ELISA with BD OptEIA TMB substrate (BD Biosciences). 1 M hydrosulfuric acid was used for quenching. Absorbance was read at 450 nm using a BioTek Epoch 2 microplate spectrophotometer and antibody concentrations for each sample were interpolated from the standard curve.

### IL-1β Quantification by Cytometric Bead Array Assay

Duodenum and jejunum samples were prepared by homogenizing weighed tissue in 1 mL PBS containing protease inhibitors (cOmplete™ Mini Protease Inhibitor Cocktail; Roche) using a Bead Mill 24 (Fisher Scientific). Homogenates were centrifuged at 13,000 rpm for 10 min, supernatant was collected, and the centrifugation step was repeated. Resulting supernatants were stored at −80°C prior to analysis. IL-1β levels were measured using a mouse IL-1β flex set (BD Biosciences) according to the manufacturer's protocol. Samples were analyzed using a CytoFLEX Flow Cytometer and CytExpert software (Beckman Coulter).

### Lamina Propria Cell Isolation and Flow Cytometric Analysis

A mouse LP dissociation kit (Miltenyi Biotec) was used to isolate cells from the small intestine according to the manufacturer's protocol, with a modification at the final dissociation step where cells were gently crushed through a 70 μm cell strainer immediately following enzymatic digestion to generate a single cell suspension. Cells were centrifuged for 10 min at 1,500 rpm and the cell pellet was resuspended in PBS containing 0.5% BSA. Cells were counted using a hemocytometer prior to staining. Cells were stained with fixable viability dye eFluor506 (ThermoFisher) and Fc receptors were blocked with anti-mouse CD16/32 (2.4G2; BD Biosciences). Surface staining included rat anti-mouse CD45 (30-F11; BD Biosciences), rat anti-mouse B220 (RA3-6B2; BD Biosciences) and rat anti-mouse Siglec F (E50-2440; BD Biosciences). Cells were fixed and permeabilized using BD CytoFix/CytoPerm (BD Biosciences) and incubated with rat anti-mouse IgA (C10-1; BD Biosciences). Liquid counting beads (BD Biosciences) were added to samples prior to acquiring data on a CytoFLEX flow cytometer (Beckman Coulter) to enable total cell counts of cells falling within IgA^+^B220^−^CD45^+^ live gates according to manufacturer's protocol. Data were analyzed using CytExpert software (Beckman Coulter).

### Statistical Analysis

Each data set was first analyzed for normality using a D'Agostino-Pearson omnibus normality test and a Shapiro-Wilk normality test. An unpaired *t*-test was used to assess differences between two groups of normally distributed data. A Mann-Whitney test was used to assess differences between two groups of data that were not normally distributed. A Wilcoxon test was used to assess differences between paired data sets that were not normally distributed. A *p*-value of ≤ 0.05 was considered statistically significant.

## Results

### Eosinophils Are Not Required for the Maintenance of Steady-State Secretory or Circulating IgA Levels

**Figure 1:**
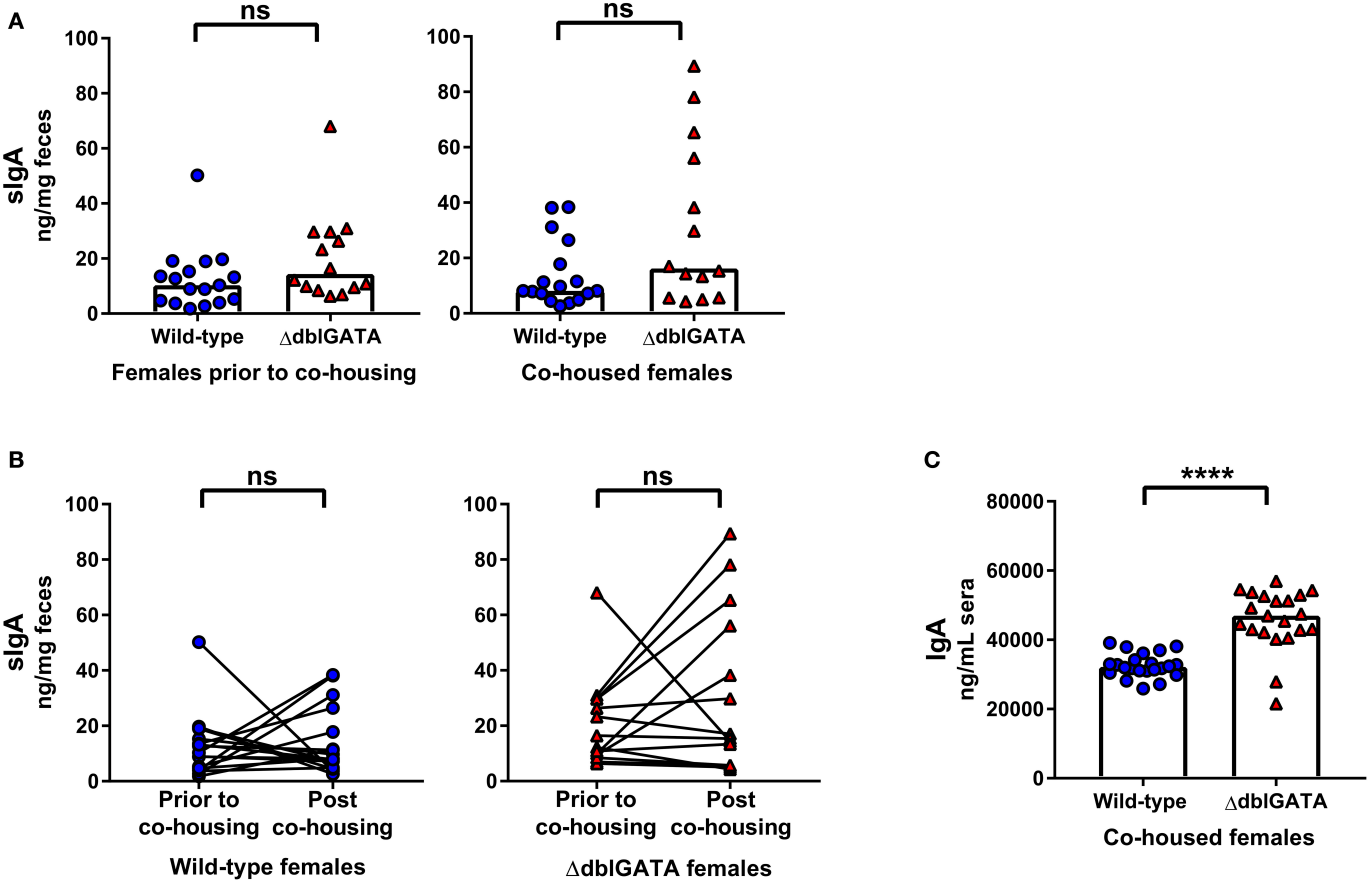
No impairment in steady-state IgA levels in female ΔdblGATA mice prior to or following co-housing with wild-type mice. Feces was collected from 6 – 11 week old female wild-type and ΔdblGATA BALB/cJ mice prior to and following 1 week of cohousing (“co-housed”). Serum was collected following co-housing. Fecal sIgA and serum IgA levels were quantified by ELISA. **(A)** Fecal sIgA data shown are pooled from four independent experiments, with a combined *n* = 14–17 in each group. Column heights are at the median value for each group and data were analyzed by a Mann-Whitney test. **(B)** The same data shown in **(A)** was assessed for the effect of co-housing on sIgA in individual mice using a Wilcoxon test on paired (pre- and post-cohousing) samples. **(C)** Serum IgA data shown are pooled from four independent experiments, with a combined *n* = 21–22 in each group. Column heights are at the median value for each group and data were analyzed by a Mann-Whitney test. Each data point is representative of an individual mouse. ns, no statistical differences; **** = *p* ≤ 0.0001.

Previous studies have examined the contribution of eosinophils to secretory and circulating IgA production. While some groups have reported a reduction in fecal sIgA (10), small intestinal sIgA and circulating IgA levels (9, 10) in eosinophil-deficient mice compared to wild-type mice, others have detected no differences (7, 11). We first set out to determine if eosinophils are required to maintain steady-state sIgA levels by analyzing fecal samples of female wild-type and ΔdblGATA mice. A recent study found that while fecal sIgA was significantly reduced in ΔdblGATA mice compared to wild-type mice, sIgA levels normalized following a period of co-housing wild-type and ΔdblGATA female mice in a single cage (12). Therefore, we too assessed sIgA levels in female BALB/cJ wild-type and BALB/cJ eosinophil-deficient ΔdblGATA mice both prior to and following a 1 week period of co-housing to confirm these findings. Since the bacterial microbiota composition is known to influence homeostatic IgA levels (18–20), co-housing is one method used to expose each genotype to the microbiota of the other with the goal of making the intestinal microbiomes of these mice less distinct. In our animal facility, we found no significant differences in levels of fecal sIgA between wild-type and ΔdblGATA females either prior to or following co-housing (Figure 1A). When we directly compared fecal sIgA levels prior to and following co-housing, we found no significant impact of co-housing on the fecal sIgA levels of either genotype (Figure 1B). We next measured serum IgA levels and found significantly elevated circulating IgA levels in female ΔdblGATA mice compared to co-housed wild-type mice (Figure 1C).

**Figure 2:**
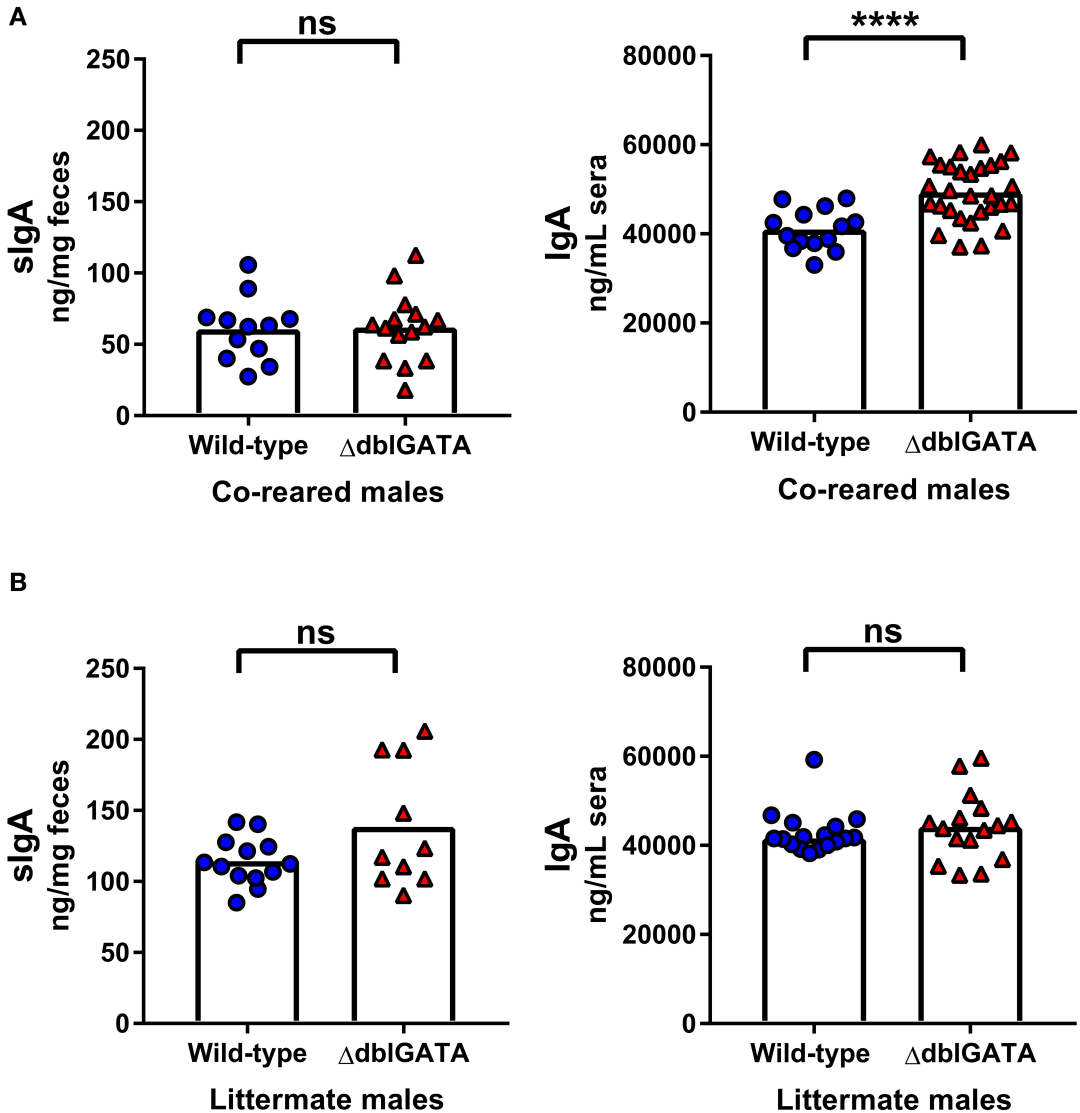
No impairment in steady-state IgA levels in male ΔdblGATA mice that were co-reared or littermates with wild-type mice. Male wild-type and ΔdblGATA BALB/cJ mice were raised in the same cage from birth but born to dams of different genotypes (“co-reared”) or raised in the same cage from birth and born to heterozygous ΔdblGATA dams (“littermates”). Feces and serum were collected when offspring were over 6 weeks old, and fecal sIgA and serum IgA levels were quantified by ELISA. **(A)** Fecal sIgA data shown are pooled from three independent experiments, with a combined *n* = 12–15 in each group. Column heights are at the median value for each group and data were analyzed by a Mann-Whitney test. Serum IgA data shown are pooled from four independent experiments, with a combined *n* = 14–29 in each group. Column heights are at the mean value for each group and data were analyzed by an unpaired *t*-test. **(B)** Fecal sIgA data shown are pooled from three independent experiments, with a combined *n* = 10–13 in each group. Column heights are at the mean value for each group and data were analyzed by an unpaired *t*-test. Serum IgA data shown are pooled from three independent experiments, with a combined *n* = 16–17 in each group. Column heights are at the median value for each group and data were analyzed by a Mann-Whitney test. Each data point is representative of an individual mouse. ns, no statistical differences; **** = *p* ≤ 0.0001.

It is not possible to co-house male mice born in separate litters and co-housing adult female mice for 1 week does not control for the potential influence of environment-induced microbiota changes during development or other extrinsic variables during development. Therefore, we set up a breeding trio that allowed us to rear ΔdblGATA and wild-type male mice in the same cage from birth: “co-reared” males (Supplementary Figure 1B). Notably, previous studies investigating the requirement of eosinophils in IgA production did not co-rear wild-type and ΔdblGATA BALB/c mice (9, 10, 13, 22, 23). We found no impairment in fecal sIgA levels in ΔdblGATA males co-reared with wild-type males (Figure 2A). Furthermore, we found no impairment in serum IgA levels in co-reared ΔdblGATA mice, in fact, we found a modest but significant increase in circulating IgA levels in co-reared eosinophil-deficient mice (Figure 1A). While most extrinsic factors that may influence IgA levels are controlled for in co-reared wild-type and ΔdblGATA mice, these mice are born to dams with different genotypes (Supplementary Figure 1B). Therefore, we set out to generate wild-type and ΔdblGATA male littermate controls by breeding a ΔdblGATA male to females that were heterozygous for the ΔdblGATA mutation (Supplementary Figure 1C). We detected no significant differences in the levels of fecal sIgA or serum IgA in male wild-type and ΔdblGATA littermate controls (Figure 2B), further strengthening our conclusion that eosinophils are not required for the maintenance of IgA under homeostatic conditions.

**Figure 3:**
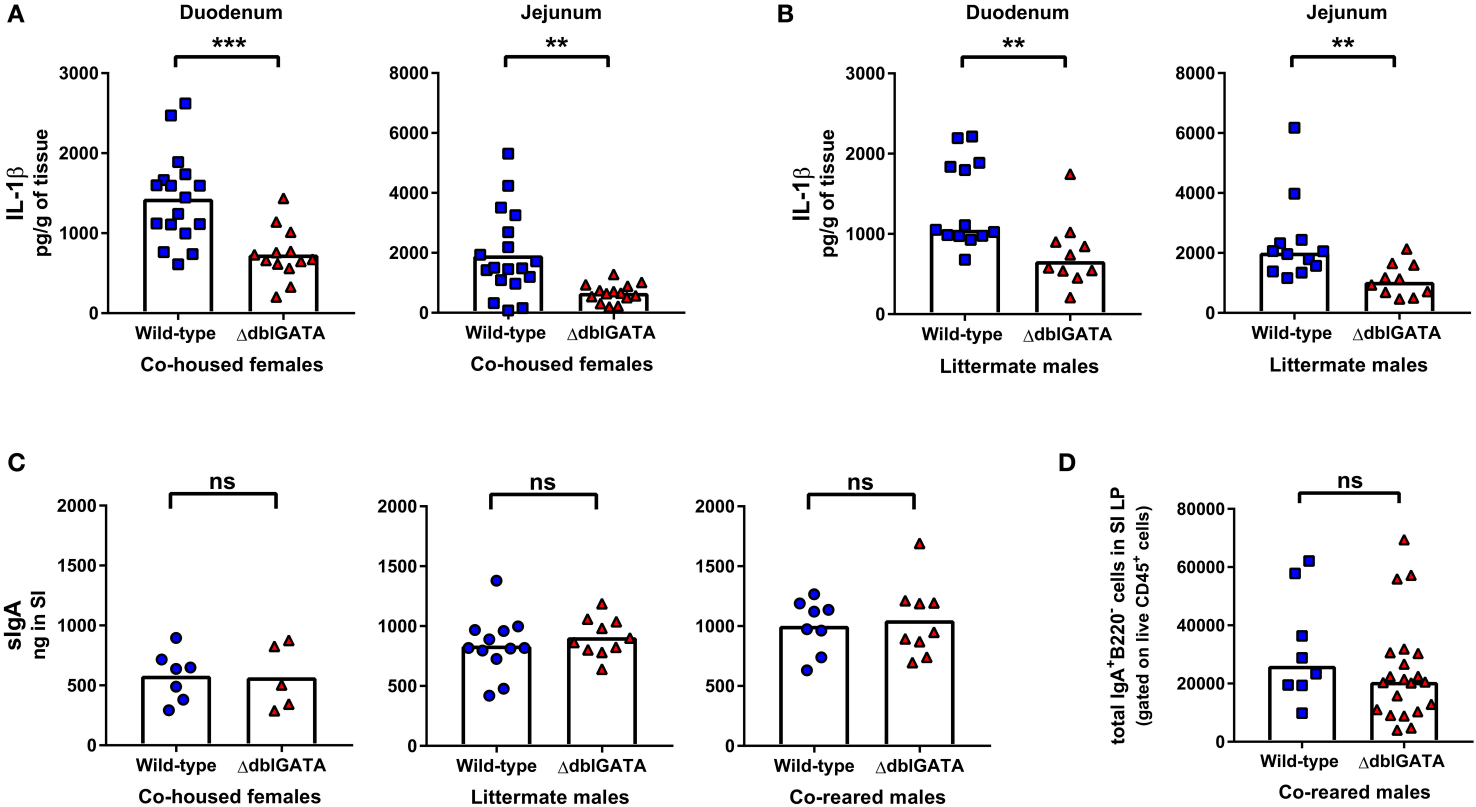
ΔdblGATA mice show reduced small intestinal levels of IL-1β compared to wild-type mice but equivalent levels of sIgA in small intestinal content. 6–11 week old female wild-type and ΔdblGATA BALB/cJ mice were co-housed for 1 week (“co-housed”) prior to collecting small intestinal tissue or content. Male wild-type and ΔdblGATA BALB/cJ mice were raised in the same cage from birth but born to dams of different genotypes (“co-reared”) or raised in the same cage from birth and born to heterozygous ΔdblGATA dams (“littermates”), and small intestinal (SI) tissue or content were collected when mice were 6–12 weeks old. **(A)** IL-1β was measured in homogenized duodenum and jejunum samples using a cytometric bead array assay. Data shown are pooled from three independent experiments, with a combined *n* = 13–17 in each group. Column heights are at the mean value for each group and data were analyzed by an unpaired *t*-test. **(B)** IL-1β was measured in homogenized duodenum and jejunum samples using a cytometric bead array assay. Data shown is pooled from two independent experiments, with a combined *n* = 10–13 in each group. Column heights are at the median value for each group and data were analyzed by a Mann-Whitney test. **(C)** sIgA was quantified in SI content by ELISA. Data shown from co-housed females are pooled from two independent experiments, with a combined *n* = 5–7 in each group. Data shown from male littermates are pooled from three independent experiments, with a combined *n* = 10–12 in each group. Data shown from co-reared males are pooled from two independent experiments, with a combined *n* = 8–9 in each group. Column heights are at the mean value for each group and data were analyzed by an unpaired *t*-test. **(D)** IgA-producing plasma cells (Live CD45^+^ IgA^+^ B220^−^cells) were quantified by flow cytometry in the small intestinal lamina propria (SI LP). Data shown are from three independent experiments, with a combined *n* = 8–21 in each group. Column heights are at the median value for each group and data were analyzed by a Mann-Whitney test. Each data point is representative of an individual mouse. ns, no statistical differences; ** = *p* ≤ 0.01; *** = *p* ≤ 0.001.

Small intestinal eosinophils express IL-1β (9, 24), and it has been proposed that baseline IgA production, seen locally in the small intestine, is supported by eosinophil-derived IL-1β signaling (9). We too found a significant reduction in IL-1β levels in the duodenum and jejunum of ΔdblGATA females that had been co-housed with wild-type mice (Figure 3A) and in male ΔdblGATA littermate controls (Figure 3B). However, when we measured local sIgA within the small intestine of either co-housed female wild-type and ΔdblGATA mice, male wild-type and ΔdblGATA littermate controls, or wild-type and ΔdblGATA males who had been co-reared we detected no differences in the levels of small intestinal sIgA (Figure 3C). To determine if an absence of eosinophils alters the population of IgA-producing plasma cells in the small intestinal LP during homeostasis, we quantified these cells in co-reared male controls by flow cytometry. Consistent with the unaltered local levels of sIgA in the small intestine of eosinophil-deficient mice, we found similar total numbers of IgA-producing cells in the small intestinal LP of co-reared wild-type and ΔdblGATA male mice (Figure 3D). Together, our data confirm that eosinophils are not essential for the maintenance of steady-state serum IgA or sIgA, nor for the maintenance of IgA-producing plasma cells in the small intestinal LP.

### Rearing Conditions Rather Than an Absence of Eosinophils Impact Steady-State Circulating Antibody Levels

**Figure 4:**
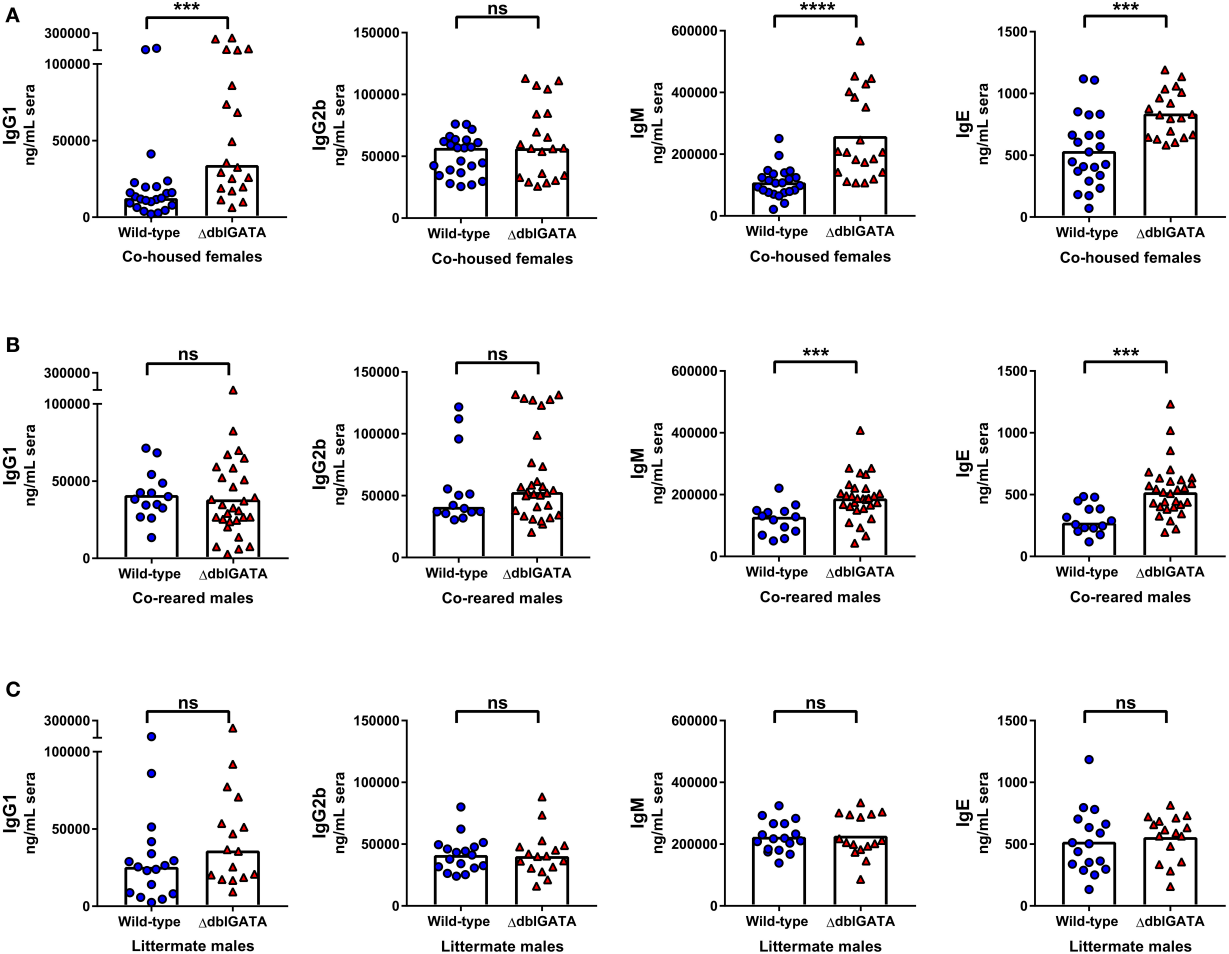
Rearing conditions rather than an absence of eosinophils impact steady-state circulating antibody levels. 6–11 week old female wild-type and ΔdblGATA BALB/cJ mice were co-housed for 1 week (“co-housed”) prior to collecting sera. Male wild-type and ΔdblGATA BALB/cJ mice were raised in the same cage from birth but born to dams of different genotypes (“co-reared”) or raised in the same cage from birth and born to heterozygous ΔdblGATA dams (“littermates”), and sera were collected when mice were over 6 weeks old. Serum antibody levels were measured by ELISA. **(A)** Serum IgG1, IgG2b, IgM and IgE levels in co-housed female mice. Data shown are pooled from four independent experiments, with a combined *n* = 19–23 in each group. IgG1, IgG2b, and IgM data column heights are at the median value for each group and data were analyzed by a Mann-Whitney test; the IgE data column height is at the mean value and data were analyzed by an unpaired *t*-test. **(B)** Serum IgG1, IgG2b, IgM, and IgE levels in co-reared male mice. Data shown are pooled from four independent experiments, with a combined *n* = 14–29 in each group. The IgG1 data column height is at the mean value for each group and data were analyzed by an unpaired *t*-test; IgG2b, IgM, and IgE data column heights are at the median value for each group and data were analyzed by a Mann-Whitney test. **(C)** Serum IgG1, IgG2b, IgM, and IgE levels in male littermate mice. Data shown are pooled from three independent experiments, with a combined *n* = 16–17 in each group. IgG1 and IgG2b data column heights are at the median value for each group and data were analyzed by a Mann-Whitney test; IgM and IgE data column heights are at the mean value for each group and data were analyzed by an unpaired *t*-test. Each data point is representative of an individual mouse. ns, no statistical differences; *** = *p* ≤ 0.001; **** = *p* ≤ 0.0001.

We next set out to determine if an absence of eosinophils affected circulating levels of other antibody isotypes, using co-housed, co-reared and littermate wild-type and ΔdblGATA mice. Circulating IgG1, IgM and IgE levels were significantly elevated in ΔdblGATA females compared to co-housed wild-type females, whereas IgG2b levels were comparable between the two genotypes (Figure 4A). Similarly, when we assessed circulating antibody levels in co-reared male wild-type and ΔdblGATA mice, we detected a significant increase in circulating IgM and IgE in ΔdblGATA mice, whereas IgG1 and IgG2b levels were comparable (Figure 4B). Early life bacterial microbiota compositions can affect circulating IgE levels in adulthood (16, 17) and while co-reared male mice are lifelong cage mates they are born from dams with different genotypes (Supplementary Figure 1B). Therefore, we measured circulating antibody levels in wild-type and ΔdblGATA littermate controls born to mothers with the same genotype (Supplementary Figure 1C) to ensure that the microbes and other factors transferred from dam to offspring were optimally controlled for. Under these conditions we detected no difference in circulating IgG1, IgG2b, IgM, or IgE levels between wild-type and ΔdblGATA mice (Figure 4C). Together these data reveal that while extrinsic factors influenced by housing and rearing conditions can impact circulating antibody levels, an intrinsic lack of eosinophils does not affect circulating antibody levels under homeostatic conditions.

## Discussion

Tissue-resident eosinophils are being increasingly recognized for their roles in development and immune regulation under steady-state conditions (6). Eosinophils are highly abundant in the small intestine and it has previously been reported that they support the production of sIgA (9, 10), however, more recent studies have found that eosinophils are not essential for maintaining homeostatic IgA levels (7, 11, 12, 22, 23). Other environmental factors such as colonization of the intestinal tract by specific bacterial species can significantly influence IgA production (12, 18–20, 25). Therefore, taking measures to control for these extrinsic effects during development is of critical importance when quantifying steady-state antibody levels in adulthood. This report is the first to describe steady-state secretory and circulating antibody levels in BALB/cJ wild-type and eosinophil-deficient ΔdblGATA mice that were sourced from a single vendor and either co-housed, co-reared or littermates. After controlling for extrinsic variables, we found that eosinophils are not essential to maintain steady-state IgA levels.

The commercial source of mice can significantly impact their intestinal microbiota composition, therefore, using mice from a single vendor is one of several strategies available to control for environmental influences when studying mice of different genotypes (26, 27). Even with these precautions, caging effects and the impact of genotype on the microbiota can result in mice harboring differing microbiota compositions, and distinct microbiota compositions between dams has been shown to influence IgA levels in adult offspring (25). The impact of microbiota composition on IgA levels in ΔdblGATA mice has been recently demonstrated: ΔdblGATA mice that were bred in-house were found to have reduced sIgA levels compared to wild-type mice sourced from a commercial vendor, yet after a period of co-housing sIgA levels normalized between genotypes (12). This study identified small intestinal colonization by specific microbes as the extrinsic modifier of sIgA levels, rather than an intrinsic lack of eosinophils (12).

While co-housing is a simple method to make the microbiota compositions of mice born to different dams less distinct, it is not possible to co-house male mice born in separate litters and this method does not resolve the influence of unique microbiota compositions during the neonatal period when the mucosal immune system is under significant development. By co-rearing male ΔdblGATA and wild-type mice we controlled for cage-driven effects on microbiota composition. Additionally, we generated wild-type and ΔdblGATA littermates to optimally control for extrinsic factors during gestation and birth, as these mice were born from dams in the same cage and with the same genotype. Our experimental set up allowed us to conclusively confirm that an intrinsic lack of eosinophils did not impact circulating antibody levels.

Recently, a study set out to determine if the presence or absence of eosinophils influences the microbiota composition of mice using littermate controls (11). This group used male wild-type and ΔdblGATA littermates to demonstrate that a genetic loss in eosinophils intrinsically results in an altered intestinal microbiota composition (11). The most prominent distinction was reduced microbial diversity in the intestinal mucus layer of eosinophil-deficient mice, compared to eosinophil-sufficient littermates, illustrating that eosinophils themselves either directly or indirectly affect the ability of microbes to colonize the gut (11). In our opinion studies such as this and our own are critical to tease apart the effect of genotype on microbiota composition and immune functions (intrinsic effects), from environmental factors that may influence microbiota composition and immune functions (extrinsic effects). One explanation for conflicting reports in the literature on whether and how eosinophils impact steady-state IgA levels is that different groups have inconsistently controlled for extrinsic factors that may have influenced IgA levels, for example by co-rearing (or not) mice of differing genotypes. Alternatively, it is possible that discrepancies in reports are from facility to facility differences in microbial exposures to all mice being housed that could impact mucosal immune responses (27, 28). It remains possible that while our data shows eosinophils are not essential for maintaining steady-state IgA levels, eosinophils may play a role in supporting IgA production under more inflammatory conditions (22, 23).

While the focus of our present study aims to assess eosinophil function during homeostasis, the role of eosinophils in IgA production during infection has also been investigated. During intestinal parasite infections additional eosinophils are recruited to the intestinal tract (22, 23). *Toxoplasma gondii*, an intracellular apicomplexan parasite that drives inflammation in the murine small intestine induces an IgA response, yet on day 10 post-infection in ΔdblGATA mice a significant reduction in IgA^+^ cells was found in the small intestinal tissue compared to wild-type mice (22). Similarly, ΔdblGATA mice infected with the small intestine-dwelling helminth, *Heligmosomoides polygyrus* had a significant reduction in IgA^+^ B cells in the Peyer's patches 14 days post-infection (23). Interestingly, it was only during infection that both of these research groups found that a loss of eosinophils impacted IgA^+^ B cell numbers: in naïve mice, no significant differences in the frequency of IgA^+^ cells in the small intestine (22) or in the Peyer's patches (23) were identified. This observation is consistent with our findings that eosinophils are not required to maintain IgA-producing plasma cells in the small intestinal LP at steady-state. While it is important to note that the wild-type and eosinophil-deficient mice used in these studies were sourced from different vendors and were not co-housed (22, 23), together these findings suggest eosinophils recruited during inflammation may have a different role in supporting IgA production than tissue-resident populations. In the future, it would be of interest to investigate the requirement for eosinophils in supporting IgA production during other enteric infections, for example, during infection with the bacterial species *Citrobacter rodentium* and *Helicobacter pylori* which also elicit sIgA production (19, 29).

When we measured circulating antibody levels at steady-state, we identified significantly elevated levels of circulating IgA, IgM and IgE in ΔdblGATA mice that were co-housed or co-reared with wild-type mice. While these results appear to suggest that eosinophils have a regulatory function in steady-state systemic antibody production, data we acquired from wild-type and ΔdblGATA littermate controls did not confirm these findings. Our data reveal an intrinsic lack of eosinophils does not impact steady-state antibody production, and instead, confirm that mice born to dams with different genotypes even within the same cage can display different immune phenotypes. Circulating antibody levels in mice born into the same cage but to either wild-type or ΔdblGATA dams may have been influenced by the acquisition of different microbiota species from the dams, and/or by the acquisition of antibody or other factors from the dams during gestation or suckling. IgA and IgM are present in breastmilk and this source of sIgA has been demonstrated to have lifelong effects on offspring microbiota composition (30). Microbes and sIgA transferred from dam to offspring in breastmilk and during birth could be substantial sources of early life microbiota diversity between wild-type mice born from a wild-type dam and ΔdblGATA mice born from a ΔdblGATA dam which occurs in both our co-housed and co-reared housing conditions. While circulating IgE has been studied in this context, where early life microbiota compositions have been shown to affect IgE levels into adulthood (16, 17) less is understood about how early life microbial factors affect lifelong circulating IgA and IgM levels. Nevertheless, our dataset exemplifies the utmost importance of controlled experimental design when studying the effect of genotype on immune phenotypes.

Over the past decade the requirement for eosinophils to support bone marrow-resident plasma cells has also been investigated. Initially, it was reported that secretion of the survival factors APRIL and IL-6 by eosinophils was required to maintain bone marrow-resident plasma cells (13). However more recently, and utilizing littermate controls, the absence of eosinophils was found to have no impact on plasma cell frequencies in the spleen or bone marrow of naïve mice (14, 15). The bacterial microbiota does have the capacity to drive the generation of bone marrow-resident plasma cell populations (31) and thus microbiota differences may explain conflicting conclusions between previous reports. During homeostasis, IgG^+^, IgA^+^, IgM^+^ and IgE^+^ plasma cells also reside at mucosal sites (17, 32) where they are in close proximity to eosinophils. The capacity for eosinophils to secrete plasma cell survival factors may depend on the tissue they reside in and on whether a particular immune response is being ensued; others have reported no such function for eosinophils that reside in the intestinal tract (22) and the role of eosinophils in other sites of B cell development such as the mesenteric lymph nodes has yet to be elucidated. Our data suggests that eosinophils have no critical function in supporting antibody production under steady-state conditions.

To our knowledge, our study is the first to comprehensively address the requirement for eosinophils in supporting secretory and circulating antibody levels using BALB/cJ wild-type and ΔdblGATA littermate controls originally sourced from a single vendor and bred in-house. We found that eosinophils are not required to maintain levels of sIgA and their intrinsic absence does not affect circulating IgA, IgG1, IgG2b, IgM and IgE levels under homeostatic conditions. Our findings and the conflicting published literature regarding eosinophils and regulation of antibody levels emphasize the importance of optimally controlling for extrinsic factors acting during development between mice of different genotypes.

## Data Availability Statement

The raw data supporting the conclusions of this article will be made available by the authors, without undue reservation.

## Ethics Statement

The animal study was reviewed and approved by the University of Victoria's Animal Care Committee.

## Author Contributions

RF and LR conceived the study, designed and performed experiments, analyzed and interpreted data, and wrote the manuscript. MK, KL, CG, BM, and AR performed experiments and edited drafts of the manuscript. All authors contributed to the article and approved the submitted version.

## Conflict of Interest

The authors declare that the research was conducted in the absence of any commercial or financial relationships that could be construed as a potential conflict of interest.
