# Supplementary material for: Littermate-Controlled Experiments Reveal Eosinophils Are Not Essential for Maintaining Steady-State IgA and Demonstrate the Influence of Rearing Conditions on Antibody Phenotypes in Eosinophil-Deficient Mice
Source: Front Immunol. 2020 Oct 15;11:557960. doi: 10.3389/fimmu.2020.557960 (PMC7593696; doi:10.3389/fimmu.2020.557960)
Supplement: Supplementary file 1 [file Data_Sheet_1.docx]

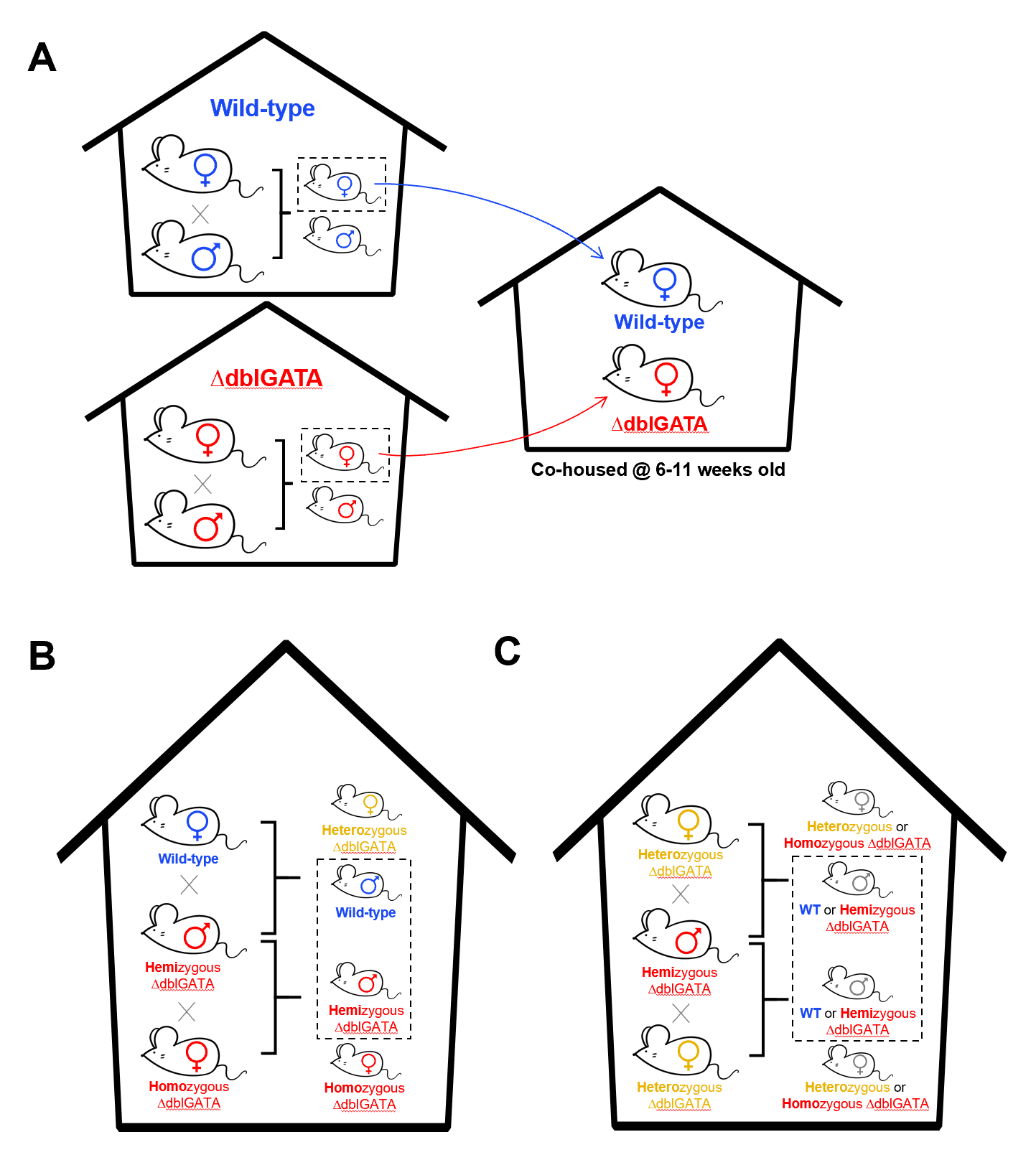
**Supplementary Figure 1. Illustration of wild-type and ΔdblGATA mice rearing schemes.**

**A.** Female wild-type and ΔdblGATA mice born in separate cages to wild-type and ΔdblGATA dams respectively were co-housed when they were 6-11 weeks old and were euthanized one week later for sample collection. **B.** ‘Co-reared’ male mice were generated by setting up breeding trios consisting of one ΔdblGATA male with one ΔdblGATA female and one wild-type female in a single cage. Male mice born to the ΔdblGATA female in this cage were all of the ΔdblGATA genotype, and male mice born to the wild-type female in this cage were all of the wild-type genotype. Co-reared male mice continued to be housed together after weaning and were euthanized for sample collection when they reached 6-12 weeks old. **C.** ‘Littermate’ male mice were generated by setting up breeding trios consisting of one ∆dblGATA male with two females heterozygous for the ∆dblGATA mutation. Male mice born to these heterozygous females were either of the wild-type or ΔdblGATA genotype. Littermate male mice continued to be housed together after weaning and were euthanized for sample collection when they reached 6-12 weeks old. As the ∆dblGATA mutation is X-linked, it is not possible to generate both wild-type and homozygous ∆dblGATA female offspring in the same cage in either the co-reared or littermate breeding schemes and therefore female offspring were not used in these experiments.

**Supplementary Figure 2. Confirmation of genotypes. A**. Representative genotyping PCR results. Lane 1: 1000 base pair DNA ladder, lane 2: no template negative control, lane 3: 459 base pair PCR product from a wild-type BALB/c mouse, lane 4: 509 base pair PCR product from a ∆dblGATA mouse lane 5: both 459 and 509 base pair products from a heterozygous female ∆dblGATA mouse. **B**. Representative flow cytometry plots showing the frequency of eosinophils (SSc^high^ SiglecF^+^ cells) amongst live CD45^+^ small intestinal lamina propria cells in mice confirmed by PCR to have wild-type (left) and ∆dblGATA (right) genotypes.
